# Clinical Manifestations of Polycystic Ovary Syndrome and Associations With the Vaginal Microbiome: A Cross-Sectional Based Exploratory Study

**DOI:** 10.3389/fendo.2021.662725

**Published:** 2021-04-23

**Authors:** Xiang Hong, Pengfei Qin, Jiechen Yin, Yong Shi, Yan Xuan, Zhengqi Chen, Xu Zhou, Hong Yu, Danhong Peng, Bei Wang

**Affiliations:** ^1^ Key Laboratory of Environmental Medicine and Engineering of Ministry of Education, Department of Epidemiology and Health Statistics, School of Public Health, Southeast University, Nanjing, China; ^2^ Department of Obstetrics and Gynecology, Medical School, Southeast University, Nanjing, China; ^3^ Department of Obstetrics and Gynecology, Zhong Da Hospital, Southeast University, Nanjing, China

**Keywords:** polycystic ovary syndrome, vaginal microbiome, clinical manifestations, testosterone, *Lactobacillus*

## Abstract

**Background:**

Previous studies suggest that the vaginal microbiome is associated with polycystic ovary syndrome (PCOS). However, the clinical manifestations of PCOS are heterogeneous. Whether the vaginal microbiome is related with different clinical symptoms was unknown.

**Materials and Methods:**

In this cross-sectional study, 89 female patients with PCOS admitted to Zhongda Hospital (Nanjing, China) were included. Basic demographic information, health-related behaviors, clinical manifestations and sex hormone levels were comprehensively recorded for all patients. Vaginal swabs were acquired for microbiota sequencing of the V3–V4 region of the 16S rRNA gene.

**Results:**

The prevalence of bacterial vaginitis and vulvovaginal candidiasis was 15.7% and 13.5%, respectively, within the PCOS patients, which were the most important factors affecting the vaginal microbiome (permutational multivariate analysis of variance test, R^2^ = 0.108, *P* = 0.001). The vaginal microbiome was associated with specific clinical manifestations of PCOS, including acanthosis nigricans, intermenstrual bleeding, pregnancy history, testosterone level and anti-müllerian hormone level, with *P* values < 0.05. The abundance of *Lactobacillus crispatus* was higher (*P* = 0.010) while that of *Lactobacillus iners* was lower (*P* = 0.036) among PCOS patients with elevated testosterone levels. Other potential bacterial biomarkers were not statistically significant after adjusting for confounding factors. No evidence of associations of other common manifestations of PCOS, such as obesity and acne, with the vaginal microbiome was obtained.

**Conclusion:**

Vaginal bacterial species among PCOS patients with variable clinical manifestations, especially differences in testosterone levels, are distinct. Further studies are essential to investigate the microbiota and molecular mechanisms underpinning this disease.

## Introduction

Polycystic ovary syndrome (PCOS) is one of the most common causes of infertility with ~10% global prevalence (1). Due to the unclear etiology of PCOS, it is necessary to explore the potential influencing factors to develop effective therapeutic strategies (2). The most common clinical symptoms include hyperandrogenism, oligomenorrhea, and polycystic ovarian morphology, often accompanied by obesity and acne (3). Clinically, different treatments are recommended for various symptoms. Therefore, efforts to develop precise prevention and intervention approaches should take into account the complexity of PCOS mechanisms.

Based on improved next-generation sequencing (NGS) technologies, our group initially identified associations between the vaginal microbiome and PCOS. The relative abundance of *Lactobacillus crispatus* (*L. crispatus*) in the PCOS patient group was significantly lower while that of *Mycoplasma* and *Prevotella* was higher than that in the control group (4). In the same year, comparable findings were reported by other research groups (5). However, these earlier studies were a case-control design, with heterogeneous manifestations among patients in the case group. For instance, a fraction of patients were positive for hyperandrogenism while the rest were negative (5). Owing to the limitations of small sample sizes, evidence on potential associations between the vaginal microbiome and different clinical manifestations of PCOS is still lacking.

A study by Wang *et al.* (6) using a letrozole-induced rat PCOS model supports the existence of a sex steroid hormone-microbiota-inflammation axis and critical roles of the vaginal and gut microbiomes in PCOS development. Population-based studies to date have focused on the hormonal changes during menopause and vaginal microbiota structure changes (7) but not in PCOS patients. The success of vaginal microflora transplants in some cases (8) has facilitated significant interest in analysis of the vaginal microbiome, with the aim of establishing effective clinical interventions (9). In the current study, we comprehensively explored the potential associations between common clinical manifestations of PCOS and the vaginal microbiome, and identified vaginal bacterial biomarkers for PCOS with variable symptoms.

## Subjects and Methods

### Study Population

From March 2019 to May 2020, 89 PCOS patients undergoing medical interventions at the reproductive clinic of Zhongda Hospital (Nanjing, China) who met the specific criteria described below were included for study. PCOS diagnosis followed the current *Chinese Guidelines for the Diagnosis of PCOS* (10) based on improved Rotterdam criteria (11). In brief, patients (1) developing at least two out of three symptoms, including oligo- and/or anovulation (fewer than eight cycles per year or more than three months without menstruation), clinical and/or biochemical signs of hyperandrogenism (hirsutism with a modified Ferriman-Gallwey score of ≥ 5 (10) or total testosterone level > 1.77 nmol/L), and polycystic ovaries (12 or more follicles in each ovary measuring 2–9 mm in diameter and/or increased ovarian volume (>10 mL) determined from ultrasound examination), (2) aged between 20–40 years, (3) reporting no use of oral estrogen for medical problems for at least six months, and (4) with the last menstrual period at least two months before the study were included. To avoid interference with the vaginal microbiome, the following exclusion criteria were used: (a) usage of antibiotics in the previous three days (None); (b) patients who were sexually active or complained of vaginal irrigation in the previous three days (3 women); (c) menstruating patients (None); (d) those diagnosed with *Neisseria gonorrhoeae* or *Trichomonad* (1 woman); and (e) those unwilling to participate (5 women). All participants signed an informed consent form and the study was approved by the Ethics Committee of Zhongda Hospital (2018ZDSYLL072-P01). In order to further illustrate the rules between vaginal microbiome and clinical manifestations of PCOS, we also included the original sequencing data of healthy women from our previous study (4).

### Clinical Manifestations

A standard questionnaire was completed by all participants. The basic information collected included age, marital status and education level. Health-related behaviors were assessed, including tobacco exposure (active smoker or exposed to second-hand smoke every day), alcohol intake (more than once a week for any type of liquor), underwear replacement frequency (once in 1–2 days or once more than two days), pad usage habit when not menstruating (Yes/No), and condom usage habit during sexual behavior in the preceding six months. Self-reported pregnancy history and menstruation conditions, including intermenstrual bleeding and oligomenorrhea, were additionally recorded. Intermenstrual bleeding was defined as spontaneous bleeding occurring between menstrual periods, either cyclical or random, and oligomenorrhea as a period of more than 35 days without menstruation (12). Physical examination was conducted by a gynecologist. The items included height, weight, waistline and hipline measurements, in addition to records of body hair, acne and acanthosis nigricans. Body mass index was calculated as weight in kilograms divided by square of height in meters. According to Chinese standard guidelines (13), patients were classified into normal (< 23.9 kg/m^2^) and overweight/obesity (≥ 24.0 kg/m^2^) groups. In terms of waist-to-hip ratio (WHR), WHR values ≥0.8 were regarded as abnormal. Total body hair status was evaluated based on modified Ferriman-Gallwey criteria and hirsutism defined as mFG scores ≥ 5 (14).

### Biochemical Parameters

Serum samples were collected in the early follicular phase (days 2-5) and hormonal and biochemical parameters measured the morning after overnight fasting, including follicle-stimulating hormone (FSH), luteinizing hormone (LH), total testosterone, sex hormone binding globulin, prolactin, anti-müllerian hormone (AMH), fasting glucose and fasting insulin. Biochemical analyses were performed in the hospital laboratory. The chemiluminescent immunoassay method was applied to measure indices using the DXI800 Immune Analyzer of Beckman Coulter Inc (CA. USA). According to laboratory standards, indices were divided into two groups, specifically, normal and elevated (**Supplementary Table S1**).

### Vaginal Swab Collection and Microenvironment Assessment

Two vaginal swabs were collected from each patient by gynecologists using standard operating procedures. Patients were placed in a lithotomy position, following which sterile swabs were used to scrape secretions at the posterior fornix with the aid of a speculum. Swabs were rotated three times to uniformly scrape any discharge. Vaginal pH was assessed using pH indicator paper. Smear microscopy examinations were completed by a professional member of staff to evaluate vaginal cleanliness grading according to the Chinese standards (details in **Supplementary Table S2**). Grades I and II were regarded as normal and grades III and IV as disordered. Meanwhile, bacterial vaginitis (BV), vulvovaginal candidiasis (VVC) and non-vaginitis were distinguished based on smear microscopy examinations according to Chinese standards (**Supplementary Table S3**). The second swab was stored in a dry tube with a unique identification number and immediately placed in a 4°C collection box. Swabs were stored at −80°C within 8 hours for subsequent nucleic acid extraction.

### Microbiome DNA Extraction and 16S rRNA Gene Sequencing

The detailed procedures for DNA extraction and 16S rRNA gene sequencing have been described previously (4). In brief, PBS buffer was used for elution of microorganisms in the swabs and the TIANamp Bacterial DNA Kit (Tiangen Biochemical Technology, Beijing, China) employed to extract and purify nucleic acids. The V3-V4 region of the 16S rRNA gene was amplified *via* polymerase chain reaction (PCR) with universal primers (338F: 5’-ACTCCTACGGGAGGCAGCA-3’, 806R: 5’-GGACTACHVGGGTWTCTAAT-3’). A library was constructed and amplified products sequenced on an Illumina HiSeq 2500 platform (Beijing Biomarker Technologies Co. Ltd. Beijing, China). According to sample collection times, we sequenced two batches at different times (39 samples on October 2019 and 50 samples on May 2020).

Sequencing data were processed using a standard procedure. Paired-end reads were merged with FLASH (v1.2.7, http://ccb.jhu.edu/software/FLASH/) and Trimmomatic software (v0.33) was used to remove tags of low quality (with more than six mismatches compared to the primers, average quality score < 20 in a 50 bp sliding window or shorter than 350 bp). Denoised sequences were clustered using USEARCH (version 10.0), and tags with ≥ 97% similarity regarded as an operational taxonomic unit (OTU). For each representative sequence, the NCBI dataset was used to annotate taxonomic information using QIIME. The numbers of reads for each sample were normalized corresponding to the sample with the least sequence. The relative abundance of OTU was comparable among different samples. All bioinformatics analyses were completed on the Biomarker BioCloud platform (www.biocloud.org)

### Statistical Analysis

Based on OTU data, Shannon α-diversity index was calculated using mothur software (version 1.30). Higher Shannon index is associated with a more diverse and rich vaginal microbiome (15). Due to abnormal distribution, Shannon indices were presented as median and interquartile range for different groups. The non-parametric Kruskal-Wallis method was used to examine differences between groups. The binary Jaccard distance, one of the β-diversity indices that focuses on differences in taxonomic abundance profiles from multiple samples, was assessed with QIIME software. The β-diversity index comparisons between groups were directly presented using Principal Coordinate Analyses (PCoA) plots. Next, the permutational multivariate analysis of variance (PERMANOVA) method was used to adjust for potential confounding factors with the R package *vegan*. Linear discriminant analysis effect sizes (LEfSe) were evaluated to determine potential biomarkers between groups (LDA value > 4.0). Tests for differential abundance of genera were performed using covariate-adjusted zero-inflated negative binomial regression using the R package *pscl*. In cases where zero-inflated models failed to converge, the standard negative binomial was implemented as a secondary modeling strategy using the R package *MASS* (16). The relative abundance of species was logarithmically transformed and presented in scatter diagrams for visualization of differences. A two-sided *P* value ≤ 0.05 was considered statistically significant.

## Results

### Basic Demographic Characteristics and Vaginal Microbiomes

**Figure 1:**
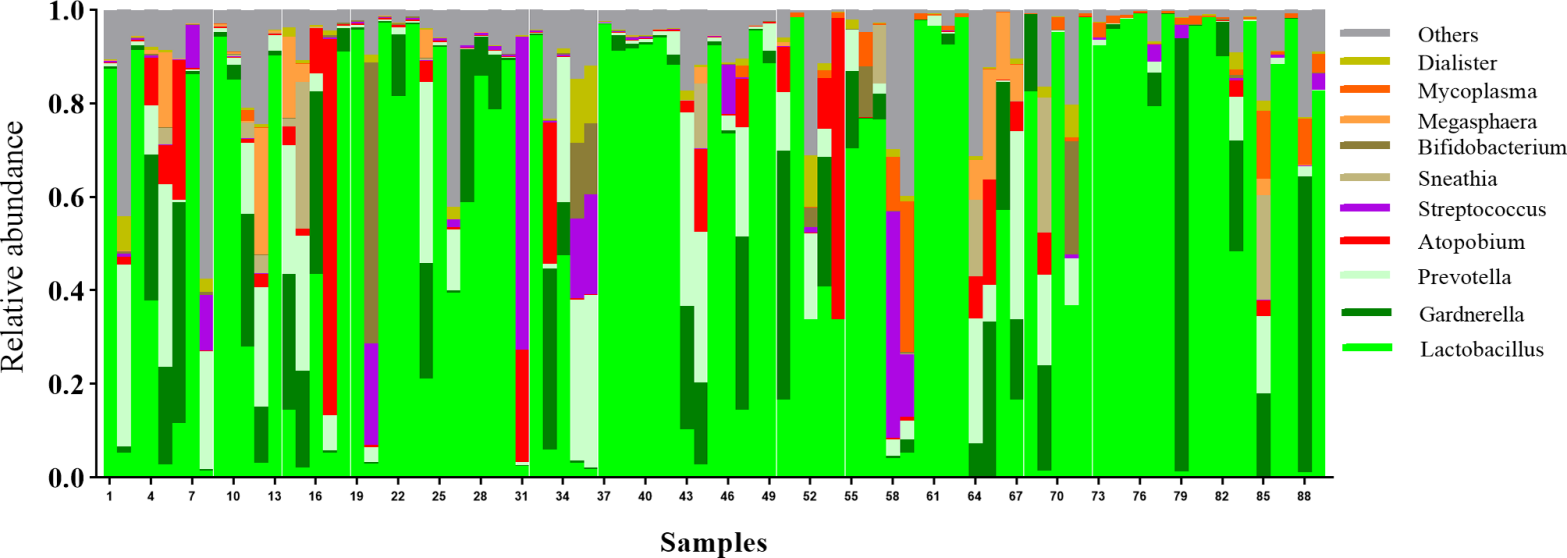
Histogram plot for relative abundance of different genera among all samples.

In total, 89 PCOS patients with an average age of 26.75 years were included for analysis. The majority of subjects were married (55/89, 61.8%), with graduate or postgraduate degrees (50/89, 56.2%) and no exposure to tobacco (56/89, 62.9%) or alcohol intake (71/89, 79.8%). Overall, 15 (17.4%) women changed their underwear once over more than two days and 23 (26.7%) had a habit of using sanitary pads when not menstruating. Twenty-three participants (25.8%) reported no sexual activity in the six-month period preceding the study. Among the remaining subjects, 48 (53.9%) women did not have a condom usage habit. The results of the vaginal microbiome sequencing are shown in the **Figure 1**, the most abundant genus include *Lactobacillus* (average relative abundance: 58.52%), *Gardnerella* (10.40%), *Prevotella* (7.94%), *Atopobium* (4.36%), *Streptococcus* (2.76%), *Sneathia* (1.57%), *Bifidobacterium* (1.55%), *Megasphaera* (1.54%) and *Mycoplasma* (1.25%). Other than condom usage, no significant associations between these factors and the vaginal microbiome were observed based on α- and β-diversity (P > 0.05). Participants that used condoms had lower vaginal microbiome diversity than those with no condom usage habit or sexual activity (Shannon index: 0.56 *vs* 1.25 *vs* 1.78, *P* = 0.017). The details are presented in **Supplementary Table S4**.

The prevalence of BV and VVC was 15.7% and 13.5%, respectively (**Table 1**). These factors were the most important determinants of the 10.8% variation in the vaginal microbiota structure (PERMANOVA test, R^2^ = 0.108, *P* = 0.001; PCoA plots are shown in **Supplementary Figure S1A**). The Shannon index was highest among women with BV (*P* = 0.001). Accordingly, adjustment of vaginitis status was considered for further multivariate analysis. Women with vaginal pH > 4.5 showed higher vaginal microbiota diversity than those with pH ≤ 4.5 (Shannon index: 1.37 *vs* 0.81, *P* = 0.006). The vaginal cleanliness grade was positively correlated with Shannon index (*P* = 0.040), signifying that bacterial diversity increases with higher grades. Our results indicate that the vaginal microenvironment could partly, but not wholly, account for the sequencing results.

**Table 1:** Vaginal microenvironment of PCOS patients and their associations with Shannon index and PERMANOVA results.

		Frequency	%	Shannon index*	PERMANOVA^&^R^2^ (*P* value)
Vaginitis	None	63	70.8	0.99 (0.95)	0.108 (0.001)
	Bacterial vaginosis	14	15.7	2.12 (0.16)	
	Vulvovaginal candidiasis	12	13.5	1.22 (1.11)	
				*P<0.001*	
PH	≤ 4.5	18	20.2	0.81 (0.53)	0.049 (0.002)
	>4.5	71	79.8	1.37 (1.36)	
				*P=0.006*	
Vaginal cleaness grading	I	6	6.7	0.80 (1.15)	0.066 (0.008)
	II	32	36.0	0.83 (0.74)	
	III	40	44.9	1.56 (1.30)	
	IV	11	12.4	1.57 (1.20)	
				*P=0.040*	

*Data are presented as median (interquartile range). The Kruskal-Wallis method was used to examine differences between the groups.

^&^PERMANOVA test performed based on binary Jaccard distance among different groups. R^2^ signifies the proportion of variation in distances that can be explained by the groups tested. P values were adjusted for sequencing batch.

### Clinical Manifestations and the Vaginal Microbiome

The main clinical features of PCOS are shown in **Table 2**. No evidence of association of the vaginal microbiome with overweight/obesity, higher WHR, hirsutism and acne was obtained (α- and β-diversity comparisons, *P* > 0.05). The prevalence of acanthosis nigricans was 32.6%, which was associated with the vaginal microbiome based on binary Jaccard distance (PERMANOVA test, R^2^ = 0.070, *P* = 0.001). In total, 28.1% participants reported intermenstrual bleeding, which was associated with richer vaginal microbiota diversity (Shannon index: 1.71 *vs* 1.06), although this difference was not statistically significant. Thirty-two participants had a history of pregnancy, which was also significantly associated with the vaginal microbiome (PERMANOVA test, R^2^ = 0.025, *P* = 0.006).

**Table 2:** Clinical features of PCOS and associations with vaginal microbiota.

Clinical features		Frequency	%	Shannon index*	R^2^ (*P* value)^&^
Overweight/Obesity	No	54	60.7	1.21 (1.25)	0.012 (0.094)
	Yes	35	39.3	1.18 (1.52)	
				*P=0.675*	
WHR	<0.8	34	38.2	1.15 (1.21)	0.019 (0.211)
	≥0.8	54	60.7	1.19 (1.31)	
	*Missing*	1		*P=0.644*	
Body hair	Normal	43	48.3	1.22 (1.08)	0.011 (0.139)
	Hirsutism	46	51.7	1.19 (1.54)	
				*P=0.736*	
Acne	No	8	9.0	0.83 (1.70)	0.010 (0.199)
	Yes	81	91.0	1.22 (1.27)	
				*P=0.315*	
Acanthosis nigricans	No	60	67.4	1.15 (1.43)	**0.070 (0.001)**
	Yes	29	32.6	1.22 (1.26)	
				*P=0.834*	
Intermenstrual bleeding	No	64	71.9	1.06 (1.10)	**0.024 (0.010)**
	Yes	25	28.1	1.71 (1.36)	
				*P=0.093*	
Pregnancy history	No	57	64.0	1.20 (1.27)	**0.025 (0.006)**
	Yes	32	36.0	1.23 (1.50)	
				*P=0.578*	

*Data are presented as median (interquartile range). The Kruskal-Wallis method was used to examine differences between groups.

^&^PERMANOVA was performed based on binary Jaccard distance among different groups. R^2^ signifies the proportion of variation in distances that can be explained by the groups tested. P values were adjusted for sequencing batch.

WHR, Waist-hip ratio. Bold value means it is statistically significant (P < 0.05).

As shown in **Table 3**, we observed no significant correlations of LH/FSH, prolactin and FBG with the vaginal microbiome (*P* > 0.05). Patients with elevated testosterone or insulin levels were likely to have richer vaginal microbiota diversity but *P* values were not significant (> 0.05). From the binary Jaccard distance comparisons, elevated testosterone, AMH and insulin were identified as contributory factors to microbiome variations (4.5%, 6.2% and 3.1%, respectively).

**Table 3:** Biochemical parameters of PCOS patients and associations with vaginal microbiota.

		Frequency	%	Shannon index*	R^2^ (P value)^&^
LH/FSH	< 2	57	64.0	1.10 (1.49)	0.006 (0.527)
	≥2	32	36.0	1.30 (0.99)	
				*P=0.791*	
Testosterone	Normal	22	24.7	0.93 (1.64)	**0.045 (0.008)**
	Elevated	64	71.9	1.23 (1.22)	
	*missing*	2		*P=0.874*	
Prolactin	Normal	78	87.6	1.20 (1.30)	0.025 (0.071)
	Elevated	7	7.9	1.20 (0.72)	
	*missing*	4		*P=0.512*	
AMH	Normal	60	67.4	1.19 (1.24)	**0.062 (0.001)**
	Elevated	11	12.4	1.03 (1.15)	
	*missing*	18		*P=0.645*	
FBG	Normal	45	50.6	1.10 (1.28)	0.018 (0.214)
	Elevated	14	15.7	1.22 (1.34)	
	*missing*	30		*P=0.521*	
Insulin	Normal	66	74.2	1.19 (1.40)	**0.031 (0.028)**
	Elevated	12	13.5	1.44 (1.25)	
	*missing*	11		*P=0.489*	

*Data are presented as median (interquartile range). The Kruskal-Wallis method was applied to examine differences between groups.

^&^The PERMANOVA test was performed based on binary Jaccard distance among different groups. R^2^ signifies the proportion of variation in distances that can be explained by the groups tested. P values were adjusted for sequencing batch.

AMH, Anti-müllerian hormone; FBG, Fasting blood glucose; FSH, Follicle-stimulating hormone; LH, Luteinizing hormone. Bold value means it is statistically significant (P < 0.05).

### Specific Genera Associated With Clinical Manifestations

**Figure 2:**
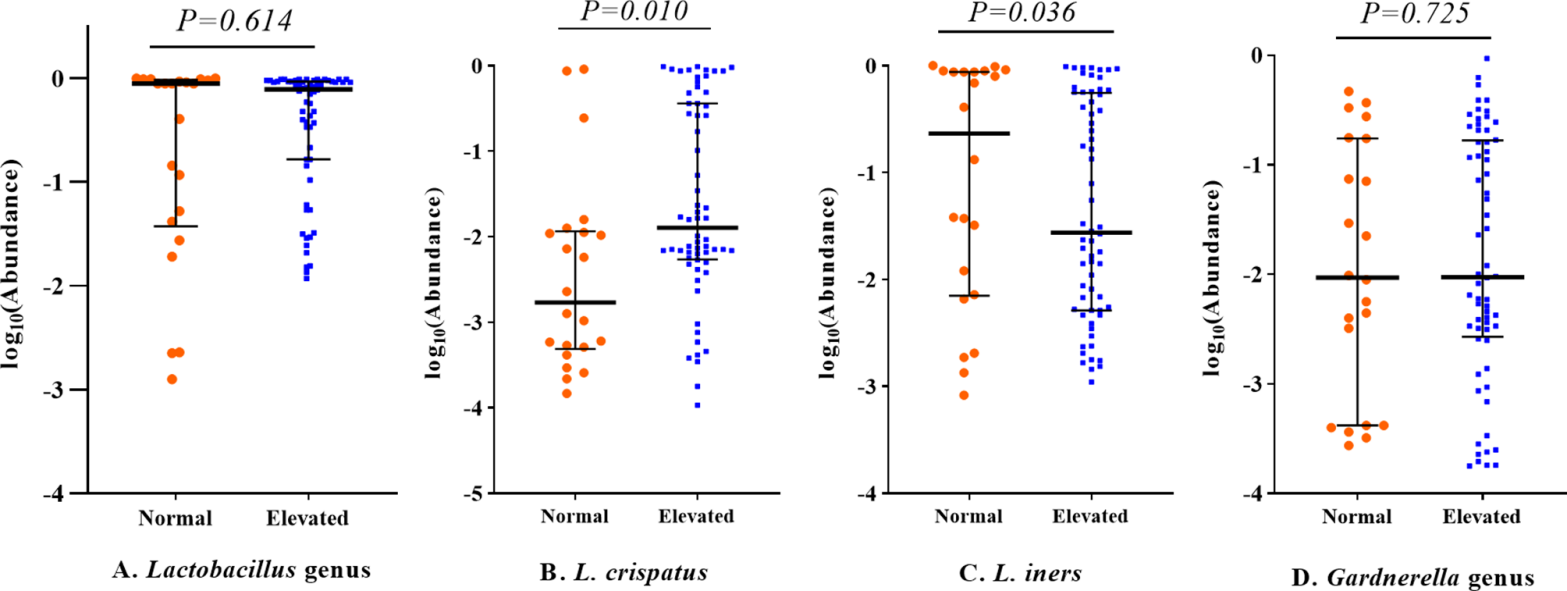
Relative abundance of *Lactobacillus* and *Gardnerella* genera among PCOS patients with different testosterone status. All *P* values were adjusted for sequencing batch, vaginitis status and condom usage during sexual activity using linear regression models. The middle lines represent the median, and the error bars represent the range interquartile. **(A)** *Lactobacillus genus*; **(B)** *Lactobacillus crispatus*; **(C)** *Lactobacillus iners*; **(D)** *Gardnerella genus*.

LEfSe analysis was further conducted for identification of potential biomarkers. The results are shown in **Supplementary Figure S2**. For confirmation of these associations, we applied zero-inflated negative or standard negative binomial models to adjust for potential confounding factors. *L. crispatus* was positively correlated with the testosterone level (β = 0.98, *P* = 0.025; **Table 4**). Other associations, including *Lactobacillus gasseri* (*L. gasseri*) and acanthosis nigricans (β = 1.10, *P* = 0.065), *Streptococcus* and intermenstrual bleeding (β = 0.29, *P* = 0.085), *Streptococcus* and pregnancy history (β = −0.99, *P* = 0.087) and *Mycoplasma* and AMH (β = 0.73, *P* = 0.194) were not statistically significant, although all LDA scores were > 4.0 in LEfSe analysis. At the genus level, abundance of *Lactobacillus* and *Gardnerella* was similar between different testosterone groups (*P* > 0.05 for all). However, at the species level, abundance of *L. crispatus* of the testosterone-elevated group was higher than that of the normal group (*P*= 0.01), while that of *Lactobacillus iners* (*L. iners*) was lower (*P*=0.036, see **Figure 2**). We further compared these genera abundance with the healthy women, who were reported in previous study (**Supplementary Figure S3**) (4). The abundance of *Gardnerella* among healthy women was lower than PCOS women, regardless of testosterone level (all P values < 0.05). Meanwhile, the abundance of *L. crispatus* of healthy group was higher than that of PCOS women, regardless of testosterone level (all P values < 0.05).

**Table 4:** Potential biomarker constituents of the vaginal microbiota for clinical features and hormone levels of PCOS patients*.

	Genera/Species	Crude β (SE)	*P*	aβ (SE)^&^	P
Acanthosis nigricans	*L.gasseri*	0.97 (0.51)	0.058	1.10 (0.59)	0.065
Intermenstrual bleeding	*Streptococcus*	0.35 (0.19)	0.061	0.29 (0.17)	0.085
	*Megasphaera*	0.37 (0.22)	0.083	0.24 (0.20)	0.224
Pregnancy history	*Streptococcus*	-1.58 (0.47)	**<0.001**	-0.99 (0.58)	0.087
Testosterone	*L. crispatus*	1.25 (0.47)	**0.008**	0.98 (0.44)	**0.025**
AMH	*Mycoplasma*	0.50 (0.59)	0.401	0.73 (0.56)	0.194

*All potential biomarkers were selected using LEfSe analysis with LDA scores > 4.0 (**Supplementary Figures 1A–E**). Estimates are coefficients (β) from zero-inflated negative or standard negative binomial models. The findings are interpreted as change in log genera/species sequencing read counts for patients with corresponding clinical features.

^&^Models were adjusted for batch of sequencing, vaginitis status and condom usage during sexual activity.

AMH, Anti-müllerian hormone; SE, standard error. Bold value means it is statistically significant (P < 0.05).

## Discussion

In this study, we showed that the vaginal microbiome is associated with a number of clinical manifestations of PCOS, including acanthosis nigricans, intermenstrual bleeding, pregnancy history, testosterone level and AMH level. The abundance of *L. crispatus* was higher among PCOS cases with elevated testosterone levels while that of *L. iners* was lower. To our knowledge, this is the first study to provide population-based evidence that the vaginal microbiome is associated with hormone levels in PCOS patients.

In terms of bacterial populations, the vaginal microbiome is a low-diversity, *Lactobacilli-* dominated community where bacteria function to maintain vaginal homoeostasis (17). Previous investigations have generally focused on the role of microbiota in vaginitis (18). With the development of detection technology, involvement of the vaginal microbiome in progression of diseases, such as preterm birth (19), infertility (20) and endometriosis (21), has been demonstrated. However, findings on the reported functions of different *Lactobacilli* species in the vaginal microbiome are inconsistent (17) and require further investigation. Notably, we observed distinct β-diversity of vaginal microbiota between PCOS patient groups with high and normal testosterone levels. At the micro-level, no differences in BV-related bacteria, such as *Gardnerella* and *Atopobium*, were evident but differences in specific *Lactobacilli* species were detected among the two groups. *L. crispatus* are often considered ‘beneficial bacteria’ (22). However, *L. iners* bacteria contain features of probiotic lactobacilli as well as vaginal pathogens (17) and an inverse association exists between *L. gasseri* and *L. iners* (23). Although the difference for their associations with hormone levels, including androgen and estrogen, remain unclear, previous studies support potential correlations. Muhleisen et al. (7) demonstrated a decrease in the *Lactobacilli* population in line with menopause-related hormonal and vaginal epithelial changes but no convincing conclusions were reached. Pregnancy is a typical process involving changes in hormone levels. A number of studies have reported alterations in the vaginal microbiome in association with pregnancy (24). While we observed an association of higher testosterone levels with elevated *L. crispatus*, this may not be beneficial, since abundance of *Lactobacillus* was lower among PCOS patients regardless of the testosterone level (5). Our findings just suggest that *L. crispatus* and *L. iners* populations are sensitive to the testosterone level among PCOS women. However, the associated mechanisms remain to be established.

Three potential pathways have been proposed to explain the association between the vaginal microbiome and female hormone levels. Initially, sex hormones affect mucosal immunity to determine the vaginal microbiome (25). Antigen presentation, cytokine production, immunoglobulin production and transport are influenced by variations in sex hormones levels (26) and vaginal mucosal immunity directly impacts the stability of the vaginal microbiome. Meanwhile, estrogen stimulates accumulation of glycogen in the vaginal epithelium, which is thought to play a major role in maintaining protective *Lactobacillus*-dominated microbiota (25). Secondly, disorder of the vaginal microbiome potentially leads to an increase in local inflammatory factors, such as interleukin-8 and tumor necrosis factor -α (27), which, along with a number of metabolites, trigger chronic systemic inflammation through the blood and lymph system, and in turn, affect the hypothalamic-pituitary-ovarian axis (28). Finally, the association between sex steroid hormones and human gut microbiome has been widely investigated. Women in the high testosterone group clearly harbor more diverse gut microbial communities (29). Interestingly, the vaginal microbiome is reported to be affected by the gut microbiome (21). Sánchez *et al.* (30) showed that the presence of PCOS is associated with longer anogenital distance in adults, which is a biomarker of prenatal androgen exposure. PCOS patients with elevated testosterone level might due to the prenatal androgen milieu, too (31). So it is a reasonable assumption that the longer anogenital distance might reduce the interaction between vaginal and rectum microbiome, which could partly explain the relative high *L. crispatus* level among PCOS patients with elevated testosterone. This is worthy of further verification.

Our results also suggest that the abundance of *Streptococcus* is potentially associated with intermenstrual bleeding and no history of pregnancy. Komiya et al. (32) demonstrated that *Streptococcus* in the gut microbiome was more abundant among infertile women. Since PCOS is a common cause of infertility (1), the issue of whether *Streptococcus* could serve as the key biomarker for PCOS patients with infertility warrants further research. Intermenstrual bleeding is a common symptom of PCOS and closely related to hormone level disorders (33). Continuous menstrual blood flow along the vaginal wall would significantly affect the microbiome, but the underlying mechanisms need further investigation. AMH is a glycoprotein hormone structurally related to inhibin and activin from the transforming growth factor beta superfamily whose key roles are growth differentiation and folliculogenesis (34). Women with PCOS often display elevated AMH levels and its potential association with vaginal *Mycoplasma* colonization was first reported in this study. The most common *Mycoplasma* species in the vagina is the opportunistic pathogen *M. hominis*, with a 20%–50% prevalence (35). Previous studies by our group support the utility of *Mycoplasma* as a potential biomarker in PCOS screening (4). Experiments with mouse models have disclosed significant effects of female sex hormones on vaginal colonization of *M. hominis* (36), but the issue of whether AMH induces *Mycoplasma* colonization remains unknown. Further studies are required to clarify this association and the detailed mechanisms.

The strengths of this study include comprehensive collection of data on clinical features, use of advanced microbiome analysis technology and statistical approaches. Multi-dimensional information on PCOS, including basic demographic characteristics, unhealthy behavior and common clinical manifestations, could aid in clarifying the associations between the vaginal microbiome and PCOS. For example, while obesity is thought to be linked with the human microbiome (37), our results did not support its association with the vaginal microbiome among PCOS subjects. Meanwhile, condom usage was identified as an important contributory factor to changes in the vaginal microbiome. In terms of the association between PCOS and infertility, a large proportion of women did not use condoms during sexual activity. This information should be collected and further assessed in future related studies. Enrichment of data facilitated adjustment for confounding factors during our exploration of the true associations using statistical approaches. While a number of significant differences were observed in LEfSe analysis, the multiple adjustment model did not support these conclusions, which highlights directions for future research.

A number of limitations of this study were inevitable. Since our study is cross-sectional in design, causal associations could not be inferred. PCOS is a progressive disease, and therefore, collection of samples before its occurrence is difficult. Moreover, the vaginal ecosystem is ecologically dynamic (38) and a single sample is not representative. Future studies should therefore involve successive multiple sample collections and cohort designs. Secondly, our sample size was limited, especially since many confounding factors were simultaneously considered. Several differences were observed, but not to a statistically significant extent, which could be attributable to the relatively small sample size. Our preliminary results provide a platform for future investigation of potential research queries. Thirdly, vaginal microenvironments were evaluated according to routine Chinese protocols not including the Nugent score, which limits comparability of our data with relevant literature. Fourthly, we just excluded the women who used antibiotics in the previous three days, which seemed a short interval, because of the impact of antibiotics on the human microbiome would be longer (39, 40). We should extend this interval appropriately in future studies. Lastly, this study mainly focused on the PCOS women, without a matched healthy control. Although we had supplemented some analysis through including healthy women data from our previous research, it still attenuated the persuasion of this study. Further works should be done to explore whether the association between vaginal microbiome and testosterone levels is consistent among healthy women.

In conclusion, significant variations in vaginal bacterial populations were recorded among PCOS patients with distinct clinical manifestations, in particular, different testosterone levels. Further research is essential to establish the microbiotal factors underpinning this disease and provide new insights that could facilitate improvement of PCOS prevention, screening and treatment strategies.

## Data Availability Statement

The datasets presented in this study can be found in online repositories. The names of the repository/repositories and accession number(s) can be found below: NCBI PRJNA699990.

## Ethics Statement

The studies involving human participants were reviewed and approved by Ethics Committee of Zhongda Hospital. The patients/participants provided their written informed consent to participate in this study.

## Author Contributions

BW, DP, and XH contributed to conception and design of the study. XH and PQ organized the database. XH and JY performed the statistical analysis. XH wrote the first draft of the manuscript. YS, YX, ZC, XZ, and HY wrote sections of the manuscript. All authors contributed to the article and approved the submitted version.

## Funding

WB is supported by the National Natural Science Foundation of China (No. 81872634). HX is supported by the Scientific Research Foundation of Graduate School of Southeast University (grant No. YBPY1983).

## Conflict of Interest

The authors declare that the research was conducted in the absence of any commercial or financial relationships that could be construed as a potential conflict of interest.
