# Supplementary material for: Clinical Manifestations of Polycystic Ovary Syndrome and Associations With the Vaginal Microbiome: A Cross-Sectional Based Exploratory Study
Source: Front Endocrinol (Lausanne). 2021 Apr 23;12:662725. doi: 10.3389/fendo.2021.662725 (PMC8104084; doi:10.3389/fendo.2021.662725)
Supplement: Supplementary file 1 [file DataSheet_1.docx]

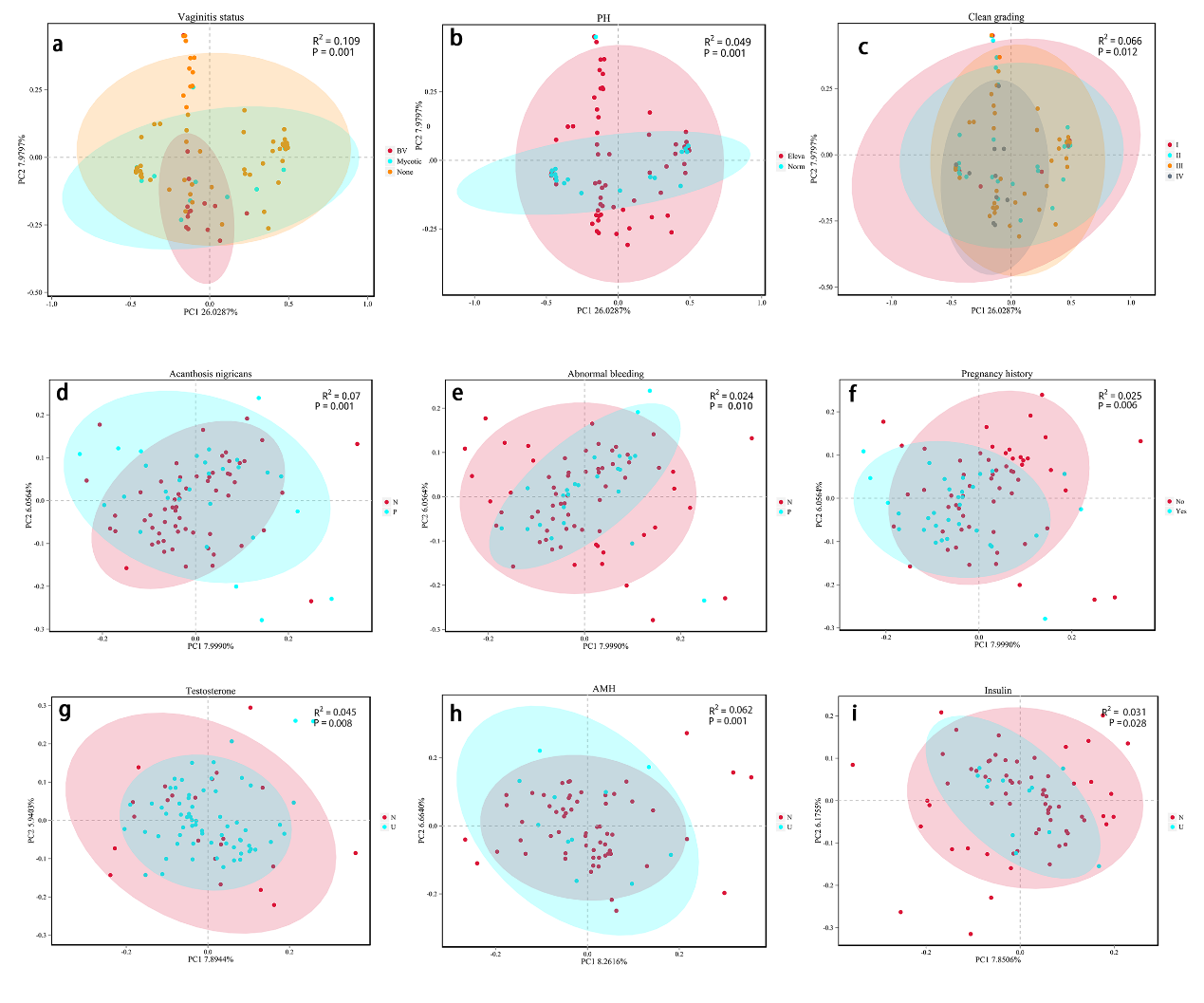


**Supplementary Figure S1. The PCoA plots for different clinical features and hormonal level.** a, vaginitis status (non, bacterial vaginitis, colpitis mycotica); b, vaginal PH (>4.5, ≤4.5); c, cleanliness grading (I/II/III/IV); d, acanthosis nigricans (N-none, P-positive); e, vaginal abnormal bleeding (N-none, P-positive); f, pregnancy history (No, Yes); g, testosterone (N-normal, U-elevated); h, AMH (N-normal, U-elevated); i, insulin (N-normal, U-elevated). Plots a-c were based on bray-curtis distance. Plots d-i were based binary-jaccard distance. All P values were adjusted for the batch of sequencing.


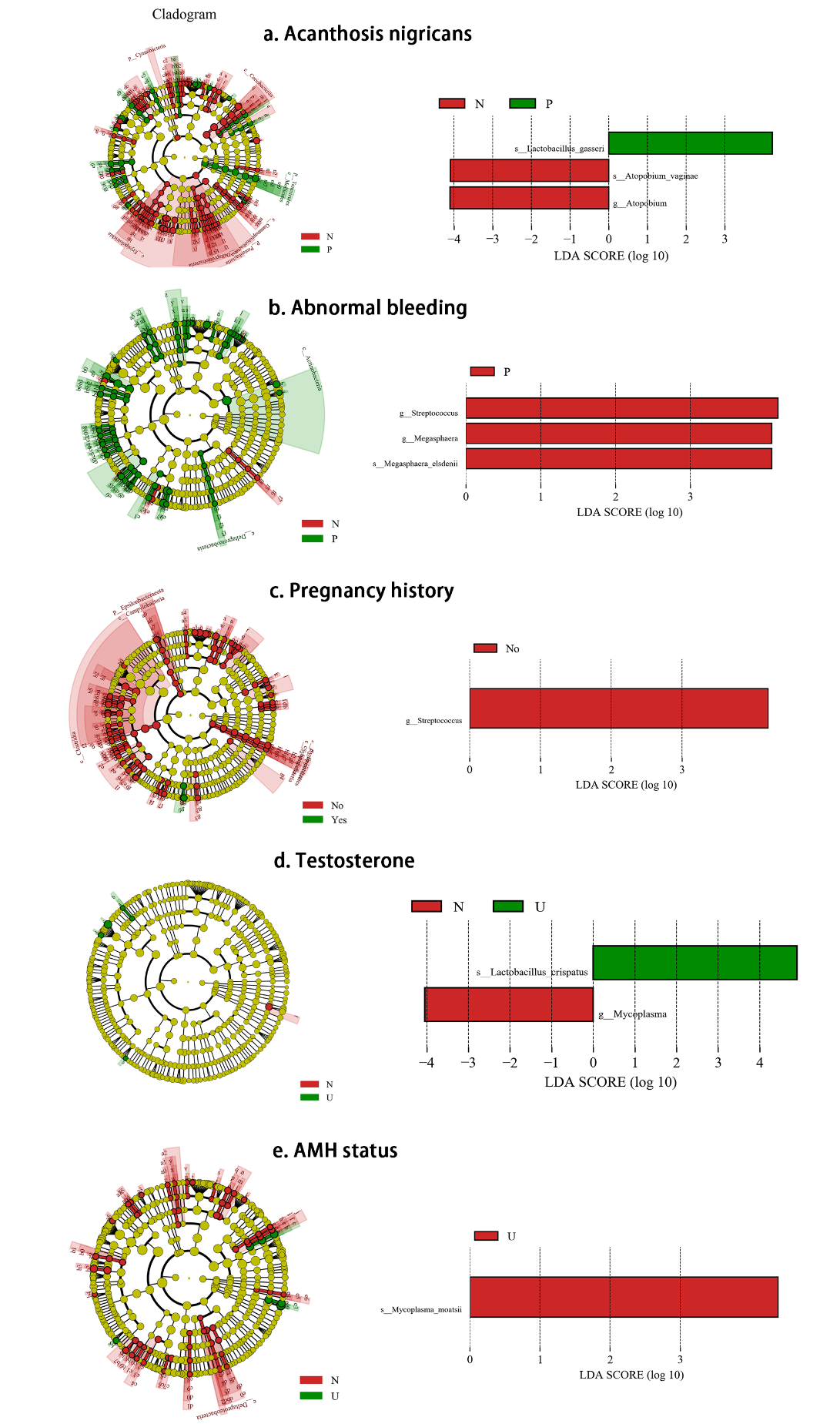


**Supplementary Figure S2. The Lefse analysis for different clinical features and hormonal level.** The plots on the left were cladograms with LDA >2.0, meanwhile, the plots on the right were the bar charts with LDA >4.0 and just show the genus and species level.


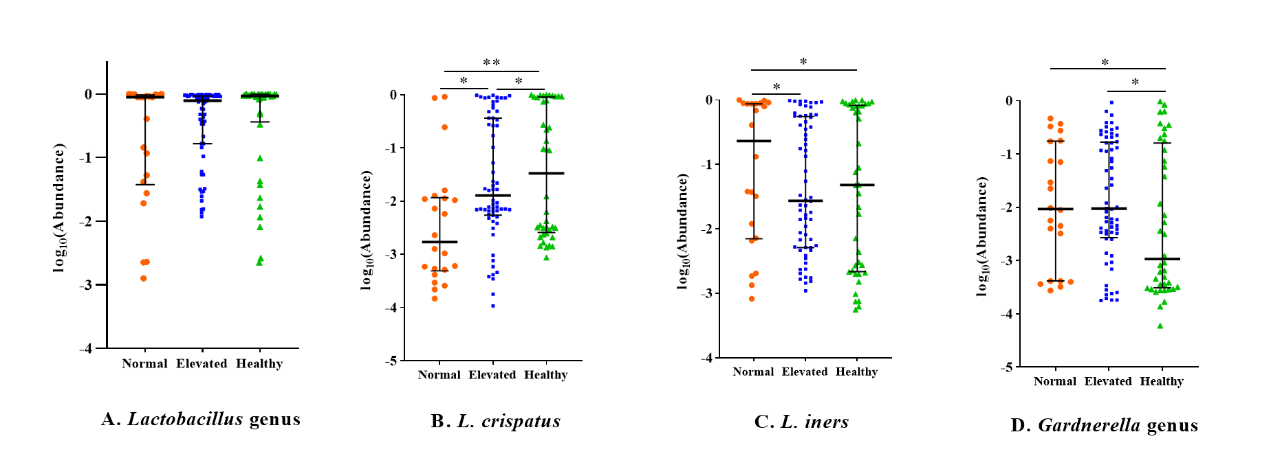


**Supplementary Figure S3. Relative abundance of *Lactobacillus* and *Gardnerella* genera among PCOS patients with different testosterone status and healthy women.** * means *P* <0.05, ** means *P* <0.001. All *P* values were adjusted for sequencing batch, vaginitis status and condom usage during sexual activity using linear regression models. The middle lines represent the median, and the error bars represent the range interquartile.
