# Supplementary material for: Clinical Manifestations of Polycystic Ovary Syndrome and Associations With the Vaginal Microbiome: A Cross-Sectional Based Exploratory Study
Source: Front Endocrinol (Lausanne). 2021 Apr 23;12:662725. doi: 10.3389/fendo.2021.662725 (PMC8104084; doi:10.3389/fendo.2021.662725)
Supplement: Supplementary file 2 [file Table_1.docx]

**Supplementary Table S1. The laboratory standard for biochemical parameters evaluation in Zhongda hospital.**

| **Parameters** | **Classification** | **Threshold value** | **Reference** |
| --- | --- | --- | --- |
| LH/FSH | < 2 | < 2 | [1] |
|  | ≥2 | ≥2 |  |
| Testosterone | Normal | ≤0.51ng/mL (1.77 nmol/L) | Laboratory |
|  | Elevated | **>** 0.51ng/mL (1.77 nmol/L) |  |
| Prolactin | Normal | ≤24.2ng/mL | Laboratory |
|  | Elevated | > 24.2ng/mL |  |
| AMH | Normal | ≤ 7.37ng/mL | Laboratory |
|  | Elevated | > 7.37ng/mL |  |
| FPG | Normal | ≤ 6.1mmol/L | [2] |
|  | Elevated | > 6.1mmol/L |  |
| Insulin | Normal | ≤ 173.43pmol/L | Laboratory |
|  | Elevated | > 173.43pmol/L |  |

AMH: Anti-mullerian hormone; FPG: Fasting Plasma Glucose

**Reference：**

[1] Endocrinology Group and guideline expert group of Chinese Medical Association Obstetrics and Gynecology Branch. Chinese Guidelines for the diagnosis and treatment of polycystic ovary syndrome[J]. Chinese Journal of Obstetrics and Gynecology, 2018, 53(1): 2-6.

[2] Alberti K G, Zimmet P Z. Definition, diagnosis and classification of diabetes mellitus and its complications. Part 1: diagnosis and classification of diabetes mellitus provisional report of a WHO consultation[J]. Diabet Med, 1998, 15(7): 539-53.

**Supplementary table S2. The vaginal cleanliness grading standard by *Chinese Expert Consensus on Clinical Application of Vaginal Microecosystem Assessment.***

| Grading | Bacteria | Cell | Health judgment |
| --- | --- | --- | --- |
| Grade I | A large number of large gram-positive rods, no other bacteria observed. | Vaginal epithelial cells, white blood cell (WBC) 0~5/HP. | Normal |
| Grade II | Some gram-positive rods, with other bacteria observed. | Some epithelial cells and pus cells, WBC 5~15/HP. | Normal |
| Grade III | A small amount of gram-positive rods, but a large number of other bacteria observed. | A large number of pus cell, WBC 15~30/HP. | Abnormal |
| Grade IV | No gram-positive rods, a large number of other bacteria observed. | A large number of pus cell, WBC more than 30/HP. | Abnormal |

**Reference:**

Infectious CMAOaGB, Group DC: Expert consensus on clinical application of vaginal microecosystem assessment. Chin J Obstet Gynecol 2016, 51(10):721-723.

Yu F, Tang Y-T, Hu Z-Q, Lin X-N: Analysis of the Vaginal Microecological Status and Genital Tract Infection Characteristics of 751 Pregnant Women. Med Sci Monit 2018, 24:5338-5345.

**Supplementary table S3. The Chinese current standards for bacterial vaginosis and vulvovaginal candidiasis diagnosis.**

| Type | Conditions | Details | Diagnosis |
| --- | --- | --- | --- |
| Bacterial vaginosis | 1. Cue cell positive | Clue cells are vaginal epithelial cells that are completely covered with non-Lactobacilli bacteria; the clue cell test is referred to as positive if > 20% of clue cells are visible on a wet mount | If three of four conditions are met. |
|  | 2. Ammonia odor test positive | The ammonia odor test was referred to as positive if there was a fishy amine odor upon the addition of 10% potassium hydroxide solution to vaginal fluid. |  |
|  | 3. Vaginal PH > 4.5 | -- |  |
|  | 4. A thin, homogeneous vaginal discharge | -- |  |
| Vulvovaginal candidiasis | 1. Yeast pseudohyphae positive | Smear microscopy showed yeast pseudohyphae. | Either of these two conditions satisfy |
|  | 2. Yeast positive | Culture method to confirm |  |

***Reference***: Ye Y, Wang Y, Shen Z. National Clinical Laboratory Procedures[M]. Nanjing: Southeast University Press, 2006.

**Supplementary Table S4. The characteristics of participants and their associations with vaginal microbiota based on Shannon index and PERMANOVA test for binary jaccard distance.**

| Characteristics |  | Frequency | % | Shannon index* | PERMANOVA^&^  R^2^ (*P* value) |
| --- | --- | --- | --- | --- | --- |
| Age, y, mean±SD | | 26.75±4.54 | |  | 0.014 (0.095) |
| Marital status | Married | 55 | 61.8 | 1.18 (1.33) |  |
|  | Unmarried | 34 | 38.2 | 1.23 (1.44) |  |
|  |  |  |  | *P=0.327* | 0.010 (0.151) |
| Educational level | Graduate and above | 50 | 56.2 | 1.22 (1.30) |  |
|  | College graduate | 20 | 22.5 | 1.02 (1.49) |  |
|  | Middle school and below | 19 | 21.3 | 1.18 (1.24) |  |
|  |  |  |  | *P=0.695* | 0.016 (0.284) |
| Tobacco exposure | No | 56 | 62.9 | 1.29 (1.48) |  |
|  | Yes | 33 | 37.1 | 1.05 (0.86) |  |
|  |  |  |  | *P=0.288* | 0.005 (0.693) |
| Alcohol intake | No | 71 | 79.8 | 1.27 (1.52) |  |
|  | Yes | 18 | 20.2 | 0.84 (0.85) |  |
|  |  |  |  | *P=0.270* | 0.016 (0.055) |
| Underwear replacement frequency | Once in 1-2 days | 71 | 82.6 | 1.20 (1.29) |  |
|  | Once more than two days | 15 | 17.4 | 1.18 (1.69) |  |
|  | Missing | 3 |  | *P=0.384* | 0.023 (0.078) |
| Pads use habit when not menstruating | No | 63 | 73.3 | 1.22 (1.22) |  |
|  | Yes | 23 | 26.7 | 0.82 (1.67) |  |
|  | Missing | 3 |  | *P=0.432* | 0.021 (0.129) |
| Condoms use during sexual behavior in recent 6 months | Without sexual behavior | 23 | 25.8 | 1.78 (1.67) |  |
|  | Nonuse | 48 | 53.9 | 1.25 (1.00) |  |
|  | Use | 16 | 18.0 | 0.56 (1.12) |  |
|  | Missing | 2 |  | ***P=0.017*** | **0.038 (0.037)** |

* The data were presented as median (interquartile range). Kruskal-Wallis method was used to test the differences between groups.

^&^ PERMANOVA test was performed based on binary jaccard distance among different groups. R^2^ means the proportion of the variation in distances can be explained by the grouping being tested. The P values represent whether or not this result was likely a result of chance.
